# Inductively Coupled Plasma-Mass Spectrometry (ICP-MS): An Emerging Tool in Radiopharmaceutical Science

**DOI:** 10.1021/jacs.4c12254

**Published:** 2024-10-31

**Authors:** Karel D. Klika, Jianlin Han, Marvin S. Busse, Vadim A. Soloshonok, Ramin Javahershenas, Frank Vanhaecke, Ata Makarem

**Affiliations:** † Molecular Structure Analysis, German Cancer Research Center (DKFZ), Im Neuenheimer Feld 280, 69120 Heidelberg, Germany; ‡ College of Chemical Engineering, Nanjing Forestry University, 210037 Nanjing, China; § Institute of Pharmacy, University of Hamburg, 20146 Hamburg, Germany; ∥ Department of Organic Chemistry I, University of the Basque Country, 20018 San Sebastián, Spain; ⊥ IKERBASQUE, Basque Foundation for Science, 48009 Bilbao, Spain; # Department of Organic Chemistry, Faculty of Chemistry, Urmia University, 57179-44514 Urmia, Iran; ¶ Atomic and Mass Spectrometry − A&MS Research Unit, Department of Chemistry, Ghent University, 9000 Ghent, Belgium

## Abstract

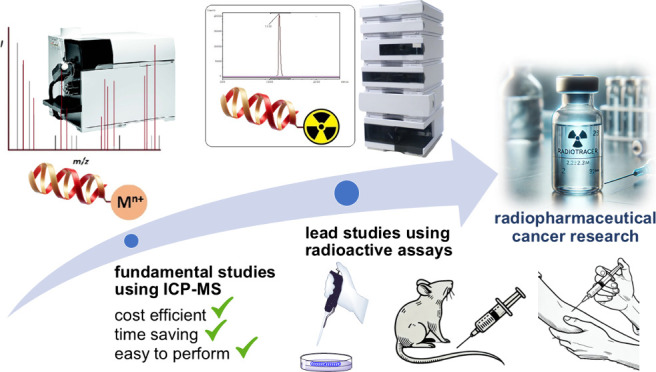

Although radioactive experiments are necessary in radiopharmaceutical drug discovery and theranostic cancer research, they are expensive, require special facilities, and face certain restrictions. Thus, finding techniques not involving radioactivity is highly beneficial for minimizing these disadvantages in such research. In this regard, methods using inductively coupled plasma-mass spectrometry (ICP-MS) have emerged as viable alternatives to traditional radioactive approaches. Despite its potential, practical applications of ICP-MS in radiopharmaceutical cancer research have only emerged in recent years. This Perspective focuses on the development and implementation of nonradioactive ICP-MS-based assays in radiopharmaceutical research and aims to inspire future research efforts in this area.

## Introduction

The utilization of radionuclides in cancer research has rapidly increased over the past decades and growth in the field of radiopharmaceutical science–dedicated to diagnostics and therapeutics (theranostics) – has been particularly strong.^1^ Presently, radiopharmaceuticals are widely used in tumor imaging (e.g., in PET scans) and also for cancer treatment via radioligand therapy.^1−5^ In this research area, radioactive assays are primarily employed to explore the bioactivity and molecular interactions of theranostic agents, both *in vitro* and *in vivo*. While radioactive approaches are important and essential tools in cancer research, they are expensive, require special facilities, and their use needs to comply with stringent regulations. Work involving radioactive compounds must be conducted at authorized institutions equipped with a radiation-controlled area and facilities which are not widely available.^6^ However, radioactive work, even at well regulated sites, exposes personnel to radiation to some extent and carries some risk of an accident. This necessitates training staff and hiring additional specialists for radioactive safety and regulatory compliance, which results in extra costs and the commitment of resources. Radioactive work also requires the proper disposal of waste and decommissioned equipment which must be done in accordance with stringent regulations. Additionally, radioassays utilizing short-lived radioisotopes (e.g., gallium-68) incur a time-dependent constraint in their operation.^7^

Hence, effective management of radioactive work is crucial to minimize costs and labor in radiopharmaceutical research. This can be accomplished by reducing the amount of radiochemistry work by employing alternative nonradioactive techniques. Positive results from nonradioactive work can then be followed up with experiments utilizing radionuclides for advanced and highly targeted studies. Thus, only truly impactful work is performed using expensive radiochemistry methods while initial exploratory work is conducted much more affordably using alternative methods. In this context, inductively coupled plasma-mass spectrometry (ICP-MS)-based assays are emerging as a promising alternative for early stage evaluation of radiopharmaceutical agents.^8−13^ ICP-MS can quantify metals, metalloids, and even some nonmetals in liquid samples at extremely low concentrations, down to ng/L or even subng/L levels.^14−16^ Utilizing ionization temperatures of ca. 7,500 K, an ICP deconstructs molecules into their constituent atoms which are subsequently ionized and then transferred to a mass spectrometer for *m*/*z*-based separation and detection. Although information on the identity of molecules is lost, the metals they contain can be detected down to ultratrace levels without requiring species-specific standards for quantification.

**Figure 1:**
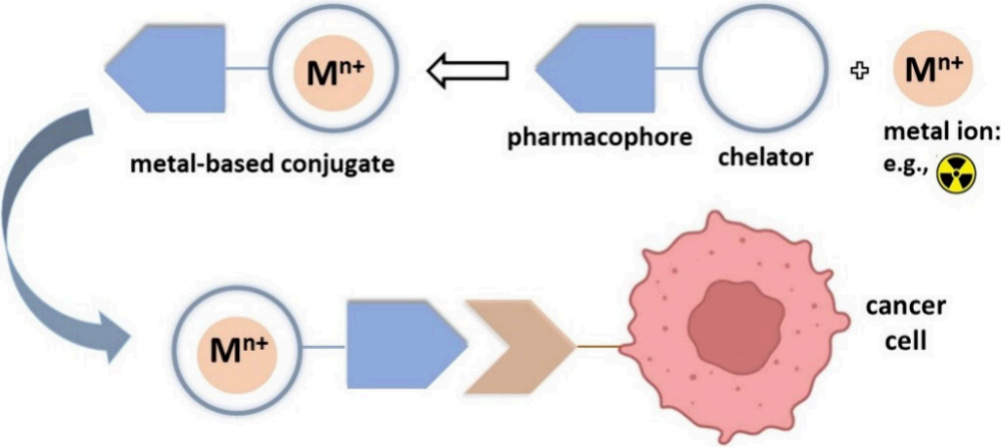
Composition and functional mechanism of typical cancer cell-targeting metal-based conjugates.

Assays using ICP-MS are therefore well-suited for detecting biomolecules containing metal ions, such as metal-based conjugates.^13,15,17−22^ Metal-based conjugates are modified bioactive molecules bearing a metal ion(s) and are currently of high interest in radiopharmaceutical drug discovery, especially for radioligand therapy.^2,3,17,23^ They structurally consist of three parts (Figure 1): a pharmacophore, which could be a small bioactive molecule, peptide, or protein; a metal/radiometal payload; and a chelator which is a small organic molecule connecting the metal to the pharmacophore.^2,24−26^

We have been investigating metal-based conjugates to develop radiopharmaceuticals and imaging probes for a number of years now.^12,23,25,26^ Numerous metal isotopes are available for use in radiopharmacy and nuclear medicine with a wide range of half-lives and diverse decay modes providing physicians with a variety of diagnostic and therapeutic options for cancer patients. Examples of popular isotopes used for preclinical and clinical purposes are listed in Table 1.^2,3^

**Table 1:** Popular Radiometals for (Pre)Clinical Applications^2,3^

radionuclide	half-life (h)	decay mode^a^	application^a^
^44^Sc	4.04	β^+^ (94%), EC (6%)	PET
^47^Sc	80.4	β^–^ (100%)	β^–^ therapy, SPECT
^66^Ga	9.49	β^+^ (57%), EC (43%)	PET
^67^Ga	78.2	EC (100%)	SPECT
^68^Ga	1.13	β^+^ (89%), EC (11%)	PET
^86^Y	14.7	β^+^ (32%), EC (68%)	PET
^90^Y	64.0	β^–^ (100%)	β^–^ therapy
^110m^In	1.15	β^+^ (61%), EC (39%)	PET
^111^In	67.2	EC (100%)	SPECT
^114m^In	1188	IT (γ emission, 97%)	Auger electron therapy
^149^Tb	4.12	α (17%), β^+^ (7%), EC (76%)	α therapy, PET
^152^Tb	17.5	β^+^ (20%), EC (80%)	PET
^155^Tb	128	EC (100%)	SPECT
^161^Tb	165	β^–^ (100%)	β^–^ and Auger electron therapy, SPECT
^177^Lu	159	β^–^ (100%)	β^–^ therapy, SPECT
^212^Bi	1.01	α (36%), β^–^ (64%)	α and β^–^ therapy
^213^Bi	0.76	α (2%), β^–^ (98%)	α and β^–^ therapy
^225^Ac	238	α (100%)	α therapy
^99m^Tc	6.02	IT (γ emission)	SPECT
^89^Zr	78.4	β^+^ (100%)	PET
^64^Cu	12.7	β^+^ (19%), β^–^ (40%), EC (41%)	β^–^ and Auger electron therapy, PET

a α, α particle; β^–^ = β particle; β^+^, positron; EC, electron capture; IT, isomeric transition; SPECT, single photon emission computed tomography; PET, positron emission tomography.

For many years now ICP-MS has been increasingly applied in various biomedical fields to quantify proteins, nanoparticles, and other substances in biological media.^27−31^ However, the first rigorous application of ICP-MS in radiopharmaceutical cancer research was only reported in 2017 by Vanhaecke and colleagues where they utilized nonradioactive ICP-MS-based assays to study the pharmacokinetics of a metal-based conjugate.^12^ Prior to this, to the best of our knowledge, there was only one preliminary study examining the use of ICP-MS for the biological evaluation of a radiopharmaceutical model,^32^ though the focus of that study was more on the analytical aspects of the technique. ICP-MS assays involving metal-based conjugates are also often used as a tool in cellular studies, such as determining receptor expression levels.^27,29,31,33,34^ Compared to fluorescent assays, evaluating metal-based conjugates using ICP-MS-based methods offers significant advantages from both chemical and pharmacokinetic perspectives. For fluorescent bioassays, the original structure of the target metal-conjugate must be modified with a fluorescent dye, thereby demanding more chemical work, time, and expense.^12^ Moreover, dye labeling can alter the pharmacokinetics of the original molecule/conjugate, especially in the case of small molecules.

ICP-MS offers a substantially higher detection power than other techniques for trace element analysis, such as atomic absorption spectroscopy (AAS) or ICP-optical emission spectrometry (ICP-OES). In addition, ICP-MS also has pronounced multielement capabilities, a wide linear dynamic range, and the capability to obtain information on the isotopic composition of the element(s) of interest.^14,15,19−22^ Straightforward use of alternative sample introduction systems further extends the application range of ICP-MS. For example, the use of laser ablation (LA) enables the direct bulk and spatially resolved analysis of solid samples with no prior digestion required while the combination of high-performance liquid chromatography (HPLC) with ICP-MS permits species-specific information to be obtained. Moreover, ICP-MS instruments are widely available in research institutes due to their extensive use in various research fields such as food chemistry and environmental science.^35^ However, like any other instrumental technique, ICP-MS has both advantages and limitations. The chief limitation of ICP-MS is when the mass analyzer has insufficient resolution to distinguish ions due to their small mass difference (referred to as polyatomic interferences and isobaric overlap). The introduction of double-focusing sector field mass spectrometers capable of high mass resolution^36,37^ – especially the introduction of collision reaction cells (CRCs) in quadrupole-based ICP-MS instrumentation^38,39^ – provided means to overcome, or at least mitigate, this limitation. When using argon as the plasma gas, for *m*/*z*’*s* ≤ 80 Da (80 Da corresponds to the argon dimer ion ^40^Ar_2_^+^), the potential occurrence of polyatomic interferences and isobaric overlap needs to be considered and accordingly addressed. Limits of detection attainable using ICP-MS vary depending on the element’s mass number, its ionization energy, and the isotopic abundance of the nuclide monitored for quantification. Given the sometimes highly dilute nature of biological samples, the limit of detection for some elements can be insufficient and thus not every assay is amenable to ICP-MS analysis.^8,15^ For γ-based radioactive bioassays, the radioactive source has no bearing on the analysis as γ-counters are unable to distinguish between radionuclides; thus assay development optimization is much reduced in comparison to ICP-MS-based methods.

In this Perspective, we examine all reported ICP-MS-based assays, both *in vitro* and *in vivo*, utilized in radiopharmaceutical science. These nonradioactive assays are used in proof-of-concept studies on the bioactivity, metabolism, and pharmacokinetics of metal-based conjugates and radiopharmaceutical models, offering a safer and more efficient option for researchers in the field. Our primary aim is to encourage researchers to develop and prioritize nonradioactive approaches in their early stage research. As stated above, although the potential applications of ICP-MS in radiopharmaceutical research have been recognized for many years,^13^ the practical application of ICP-MS in this area has only emerged in recent years. The examples reported herein come from research groups that are pioneers in the fields of radiopharmaceutical science and cancer theranostics.^8−12^ According to these reports, ICP-MS is a superior nonradioactive screening method to identify lead candidates for further investigation using radiochemistry. However, due to the limitations listed above, ICP-MS may not be applicable in all cases; for example, for fluorine-based probes as the high ionization energy of this element–which is higher than that of the plasma gas argon–precludes its efficient ionization in the ICP ion source.

## PSMA-Targeting Radiopharmaceuticals

**Figure 2:**
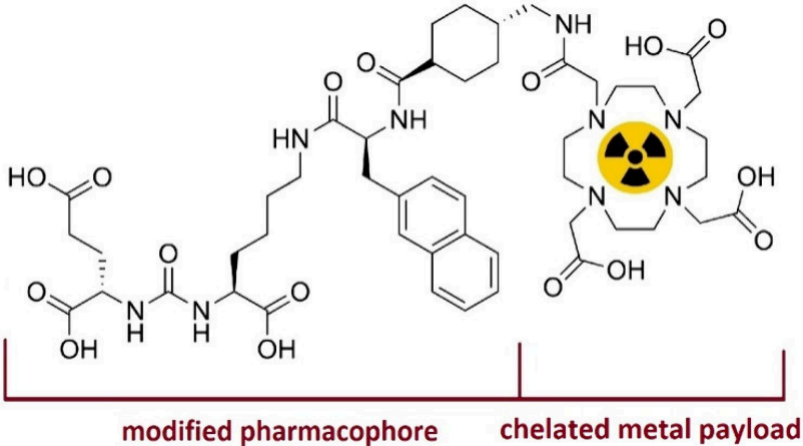
Chemical structure of metal/radiometal-labeled DOTA-PSMA-617.

Excellent examples of using ICP-MS in theranostic cancer research are the studies on DOTA-PSMA-617, both for *in vitro* and *in vivo* assays.^8−10^ DOTA-PSMA-617 is composed of a prostate-specific membrane antigen (PSMA) as the pharmacophore and a DOTA chelator for binding the metal payload (Figure 2). Lutetium-177-labeled DOTA-PSMA-617 is a popular radiodrug (known commercially as *Pluvicto*) for the treatment of advanced prostate cancer,^40−44^ and Klika, as part of the research group that reported DOTA-PSMA-617, characterized the molecular structure of DOTA-PSMA-617 by NMR.^44^

**Figure 3:**
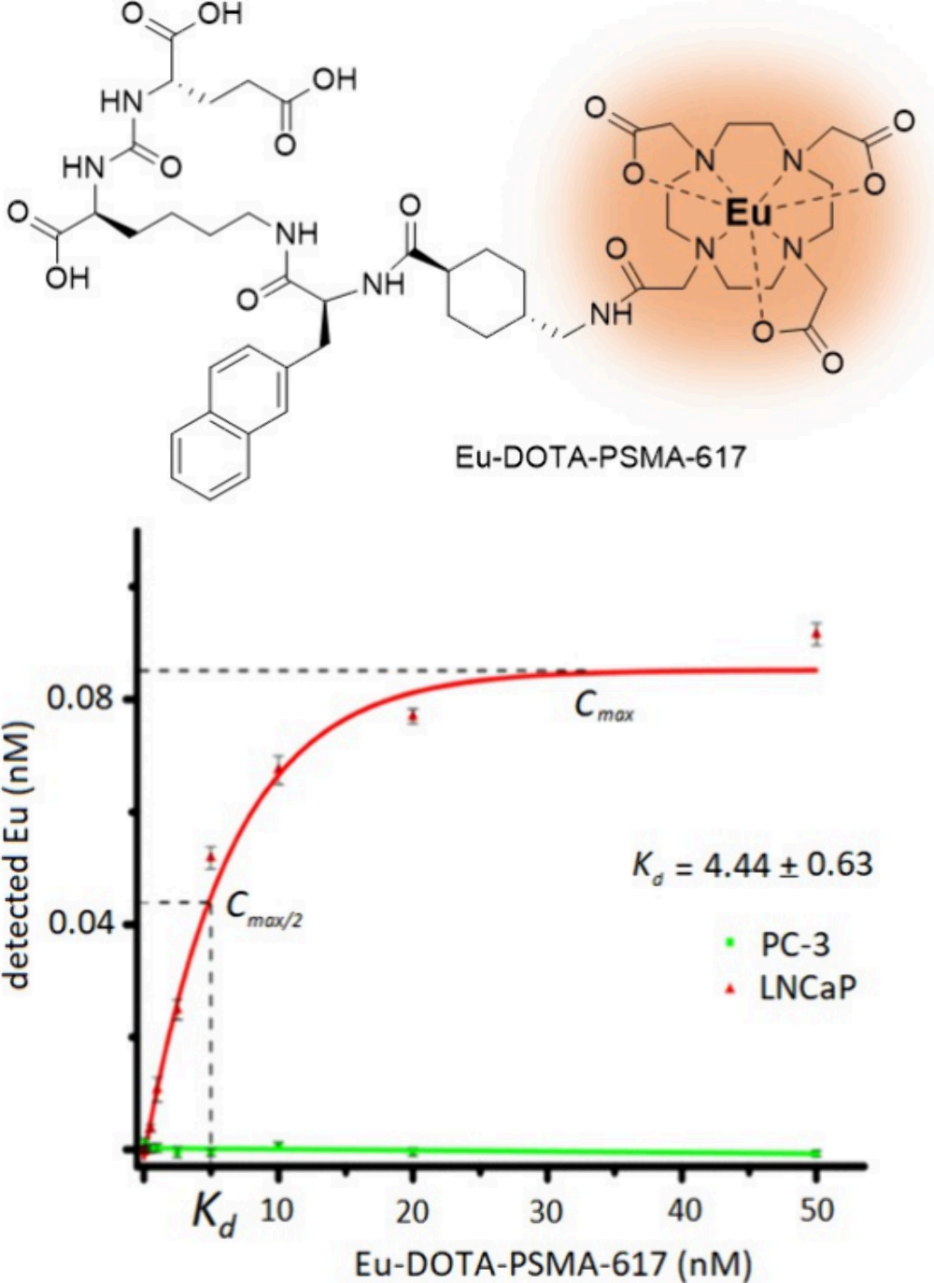
Cell binding affinity of DOTA-PSMA-617 investigated by Holzapfel et al. using ICP-MS.^8^ Adapted from Holzapfel et al., Nonradioactive Cell Assay for the Evaluation of Modular Prostate-Specific Membrane Antigen Targeting Ligands via Inductively Coupled Plasma Mass Spectrometry. *J. Med. Chem.***2019**, *62* (23), 10912–10918; copyright 2019 American Chemical Society.

In 2019, Holzapfel and co-workers developed a nonradioactive cell assay using ICP-MS to determine the binding affinity (*K*_d_) of DOTA-PSMA-617 (Figure 3).^8^ Instead of radioactive lutetium-177, they labeled the DOTA-PSMA-617 conjugate with nonradioactive europium. Europium is one of the most ICP-MS-sensitive elements with a detection limit in the ppt range (ng/L).^45,46^ As a trivalent cation, Eu^3+^ exhibits chemical properties similar to Lu^3+^ and forms strong coordination with DOTA-type chelators in a manner similar to Lu^3+^.^47^ For their assay, they employed standard cell lines in prostate cancer research, PSMA(+) LNCaP cells as a positive control and PSMA(−) PC-3 cells as a negative control.^48^ Performing a noncompetitive cellular assay, Holzapfel et al. measured a *K*_d_ of 4.44 ± 0.63 nM, which is in good agreement with the *K*_d_’s reported for DOTA-PSMA-617 in the literature.^8,43,49,50^

**Figure 4:**
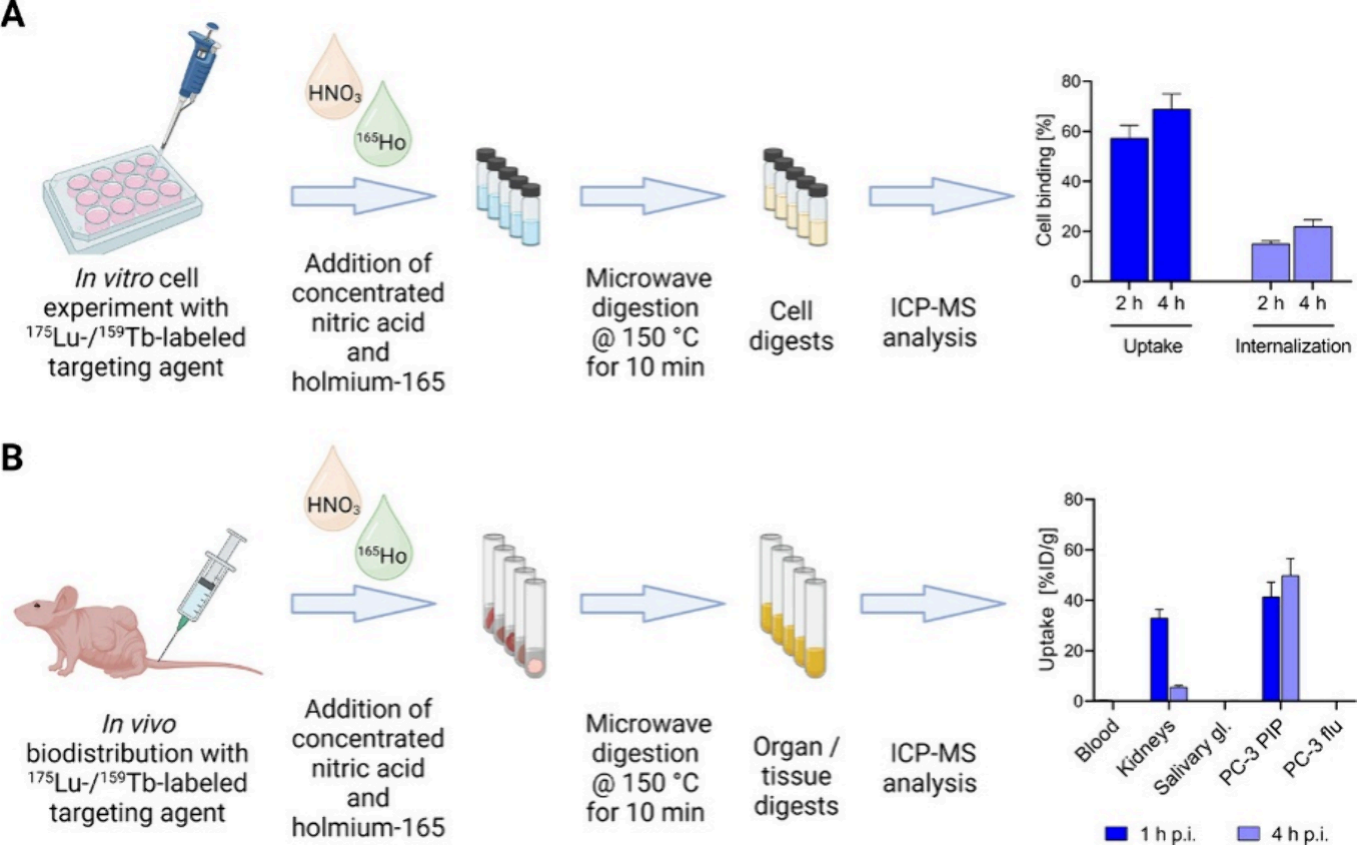
*In vitro* and *in vivo* studies on DOTA-PSMA-617 using ICP-MS conducted by Schibli et al.^10^ Reprinted from Schibli et al., Inductively Coupled Plasma Mass Spectrometry – A Valid Method for the Characterization of Metal Conjugates in View of the Development of Radiopharmaceuticals. *Mol. Pharm.***2023**, *20* (4), 2150–2158; copyright 2023 American Chemical Society.

In 2023, Schibli and colleagues conducted *in vitro* and *in vivo* studies on the bioaffinity of DOTA-PSMA-617 using ICP-MS (Figure 4).^10^ They compared ^nat^Lu- and ^nat^Tb-labeled DOTA-PSMA-617 with their respective radioactive analogs, viz. ^177^Lu and ^161^Tb. The *in vitro* uptake was similar for the nonradioactive and radioactive methods using ICP-MS and conventional γ-counting cell assays, respectively, with results differing by no more than 6%. For an ICP-MS assay using PSMA (+) PC-3 PIP cells, for ^nat^Lu-PSMA-617, *K*_d_ was determined to be 20 nM (14–28 nM) while γ-counting provided a *K*_d_ of 15 nM (12–18 nM) for its radioactive counterpart. It is worth noting that PC-3 PIP cell lines are known for their high levels of PSMA expression.^51^ Schibli et al. also conducted a biodistribution study in mice bearing PC-3 PIP and LNCaP tumors whereby ICP-MS and γ-counting techniques provided similar uptake values for the lutetium and terbium tracers in PC-3 PIP tumors. ICP-MS results from the LNCaP tumors—with lower PSMA expression compared to PC-3 PIP—revealed that lutetium can be quantified even with minimal accumulation in tissues. However, significant differences in kidney uptake were observed between the radioactive and nonradioactive mouse models, which could be due to the differences in the content of metal-labeled and nonlabeled DOTA-PSMA-617 in the injected solutions.

**Figure 5:**
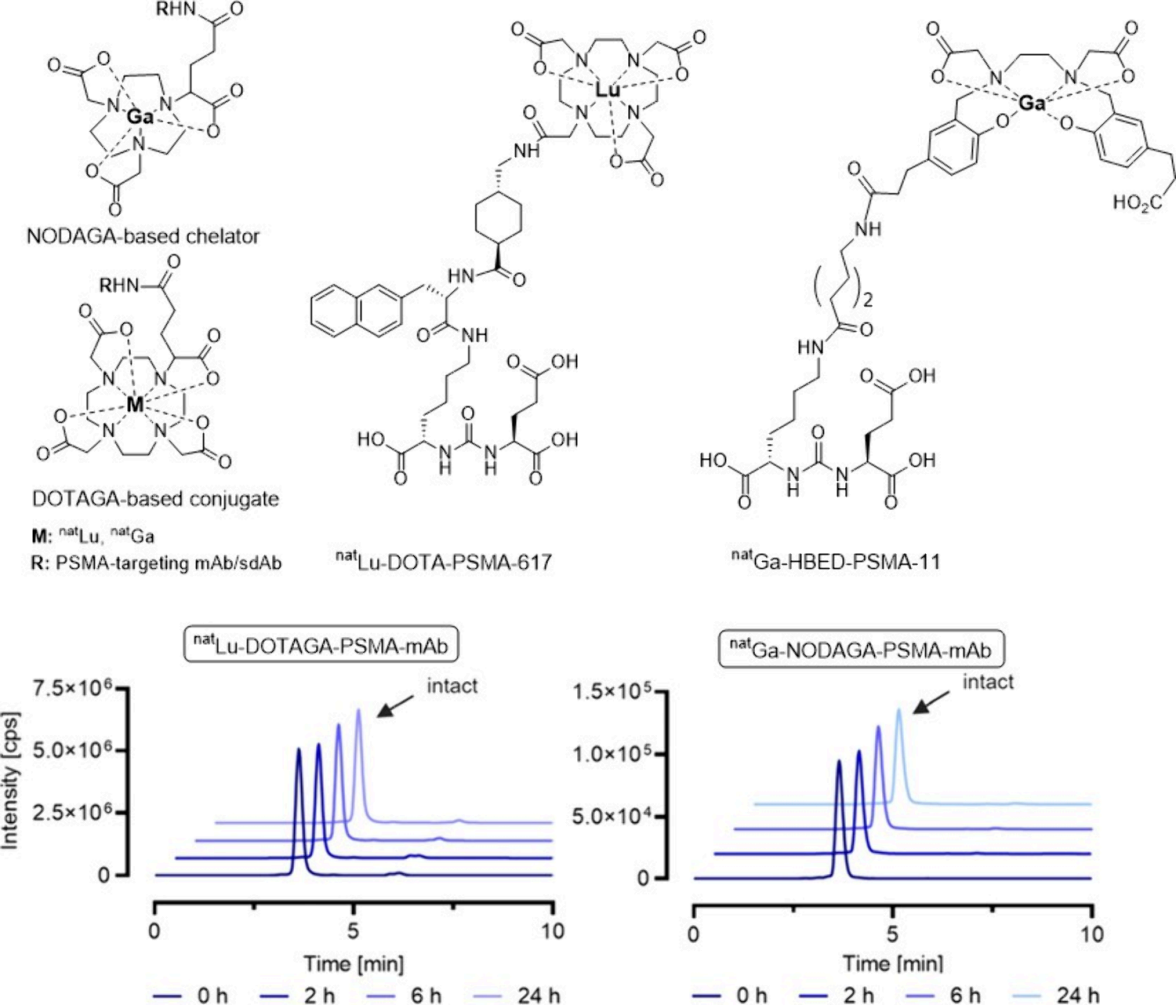
Stability studies on HBED-PSMA-11, DOTA-PSMA-617, and DOTAGA- and NODAGA-based conjugates using HPLC-ICP-MS reported by Schindler et al.^9^ Adapted from Schindler et al., Liquid Chromatography ICP-MS to Assess the Stability of ^175^Lu- and ^nat^Ga-Based Tumor-Targeting Agents towards the Development of ^177^Lu- and ^68^Ga-Labeled Radiopharmaceuticals. *Pharmaceutics***2024**, *16* (3), 299; open access under the Creative Commons CC BY 4.0 license.

Recently, Schindler’s research team used HPLC coupled to ICP-MS to assess the *in vitro* stability of several nonradioactive lutetium- and gallium-labeled PSMA-targeting conjugates, including DOTA-PSMA-617 and HBED-PSMA-11 (Figure 5).^9^ It is worth noting that ^68^Ga-labeled HBED-PSMA-11 is a widely used radiotracer in PET imaging for prostate cancer.^52−56^ Traditionally, stability studies of radiopharmaceuticals/radioconjugates in blood serum use HPLC equipped with a γ-detector to monitor the degradation process.^57^ However, in their work, Schindler et al. employed HPLC coupled to a ICP-MS. In addition to DOTA-PSMA-617 and HBED-PSMA-11, they examined the stabilities of several ^nat^Lu- and ^nat^Ga-labeled PSMA-targeting single domain antibody (sdAb) and monoclonal antibody (mAb) conjugates bearing DOTAGA^58,59^ and NODAGA^50,60^ chelators. ^nat^Lu-DOTA-PSMA-617 and ^nat^Ga-HBED-PSMA-11 were analyzed by reversed-phase liquid chromatography while the antibody metal-conjugates were analyzed by size-exclusion liquid chromatography due to their larger molecular size. The researchers did not perform any radioactive experiments themselves but instead compared their nonradioactive stability results with those reported for the radioactive counterparts in the literature.^61,62^ The stabilities for nonradioactive probes measured by ICP-MS aligned with radioactive probes containing either ^177^Lu or ^68^Ga. For example, the serum stabilities of both ^nat^Lu- and ^177^Lu-PSMA-617 at 37 °C after 24 h demonstrated >99% stability using ICP-MS and γ-counting methods, respectively.^61^

## Other Radiopharmaceutical Examples

**Figure 6:**
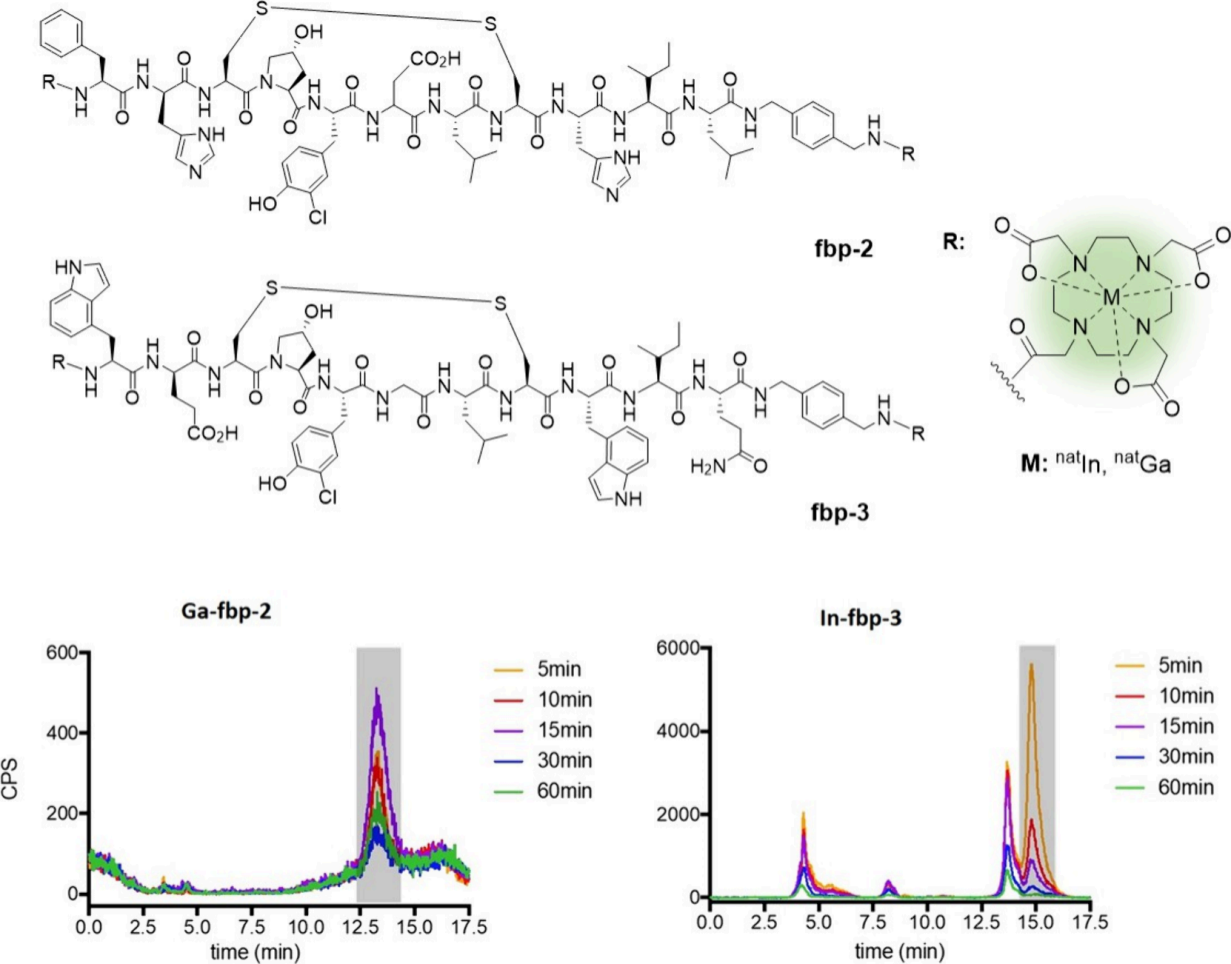
Metabolic investigation of DOTA-based peptide conjugates using HPLC-ICP-MS Reported by Caravan et al.^11^ Adapted from Caravan et al., Metabolite Profiling with HPLC-ICP-MS as a Tool for *in Vivo* Characterization of Imaging Probes. *EJNMMI Radiopharm. Chem.***2018**, *3* (1), 2; open access under the Creative Commons CC BY 4.0 license.

In another study, Caravan et al.^11^ explored the application of HPLC-ICP-MS to the metabolism of nonradioactive, metal-based conjugates using two DOTA-bearing peptide conjugates, fbp-2 and fbp-3 (Figure 6).^63,64^ The conjugates were labeled with natural abundance indium and gallium and injected into rat models. Blood samples were taken at different time points postinjection and analyzed by HPLC-ICP-MS using both reversed-phase and size-exclusion chromatography. The authors reported that the method is highly sensitive with limits of detection as low as 0.16 pmol for indium and 0.53 pmol for gallium. They were able to detect probe concentrations similar to those used in nuclear imaging studies^63,64^ with the ability to identify metabolites in concentrations as low as 0.001% ID/g. The high sensitivity of HPLC-ICP-MS also allowed them to identify *trans*-chelated byproducts and to distinguish different metabolic pathways of the probes. They also observed some differences in metabolic stabilities between the nonradioactive probes, which were similar to previously reported radioactive results.^63,64^ For example, the fbp-2 probes showed minimal degradation whereas their fbp-3 counterparts underwent relatively rapid metabolism.

Furthermore, this study demonstrated that HPLC-ICP-MS can simultaneously quantify various target metals in the one probe.^11^ This “multiplexing” capability is a significant advantage over radioactive assays as it allows multiple imaging probes to be monitored in a single animal, thereby reducing the number of animals needed for such studies. In their multielement test, gallium- and indium-labeled conjugates were injected either individually or as a mixture, and later, the concentrations of gallium and indium in the one blood sample were determined successfully.

**Figure 7:**
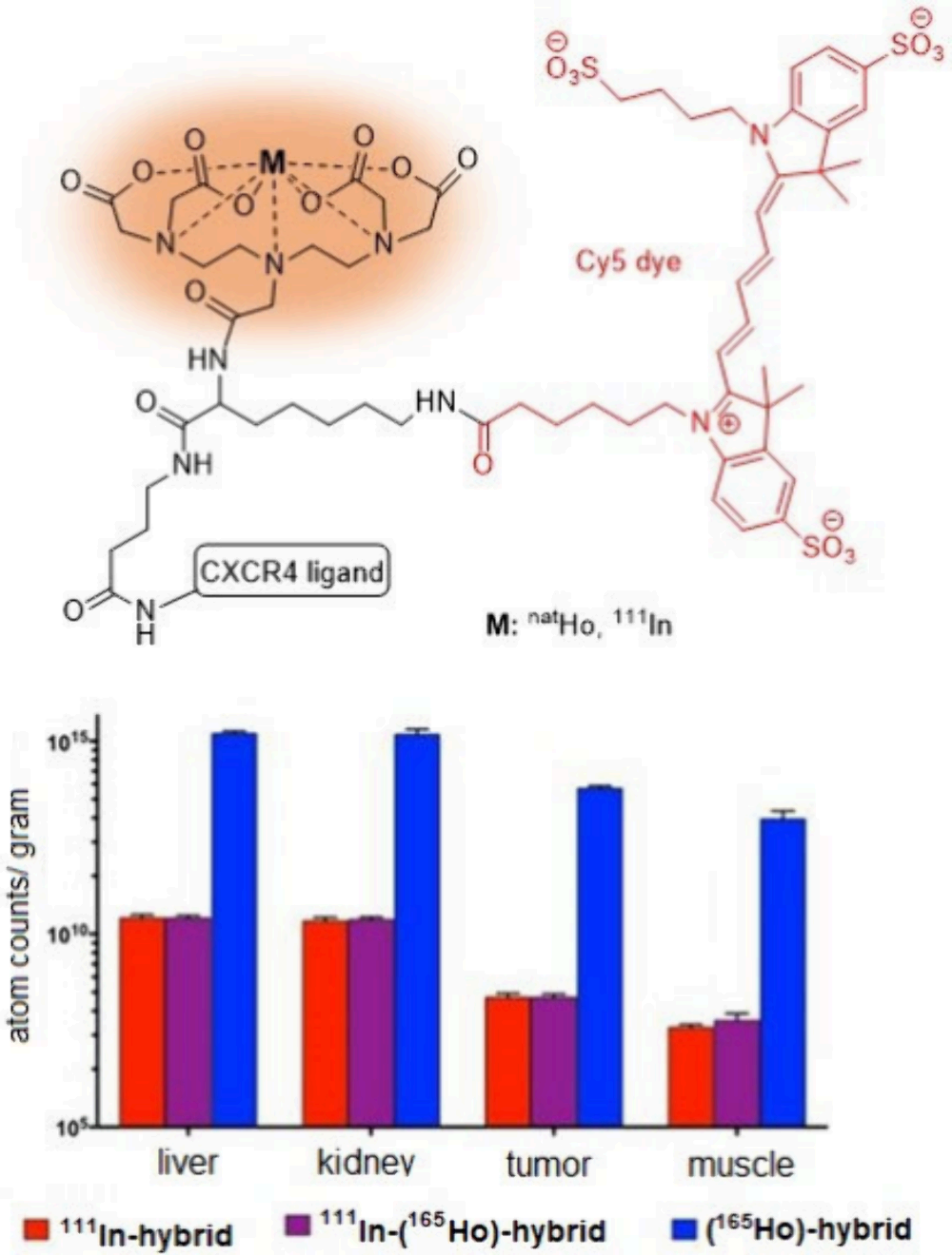
Pharmacokinetic investigation on a dual-labeled hybrid DTPA-based conjugate using ICP-MS techniques reported by Vanhaecke et al.^12^ Adapted from Vanhaecke et al., Hybrid Imaging Labels: Providing the Link Between Mass Spectrometry-Based Molecular Pathology and Theranostics. *Theranostics***2017**, *7* (3), 624–633; open access under the Creative Commons CC BY-NC 4.0 license.

In 2017, Vanhaecke and co-workers compared ICP-MS-based assays with analogous fluorescent and radioactive assays in a therapeutic investigation guided by diagnostic imaging using a hybrid molecular tracer (Figure 7).^12^ Hybrid tracers are bioconjugates labeled simultaneously with a dye as well as a metal/radiometal and are currently of high interest for use in intraoperative tumor resection surgery as dual labeling facilitates much better assessment of tracer localization in tumors.^65,66^ The hybrid tracer investigated by Vanhaecke et al. consisted of a C-X-C chemokine receptor type 4 (CXCR4)-targeting ligand,^67,68^ a Cy5 fluorescent dye,^68^ and a DTPA chelator^2^ labeled with either the stable isotope of holmium (^165^Ho) for the ICP-MS and fluorescent assays or ^111^In for the radioactive assay. The study focused on the biodistribution of this hybrid tracer in mice models bearing a human breast tumor. The mice were intravenously injected with either the nonradioactive Ho-labeled tracer, the radioactive ^111^In-labeled tracer, or a combination of both. The Ho concentration in different tissue samples was quantified by ICP-MS and the results were compared with the analogous fluorescent and radioactive approaches. In their *in vivo* study, the authors additionally employed LA-ICP-MS.^69^ In this technique, a high-energy laser is focused on the sample removing a small amount of the target material as a fine aerosol. The aerosol is then carried by a stream of carrier gas, typically helium, into the ICP. LA-ICP-MS can directly analyze solid samples and offers a spatial resolution down to ca. 1 μm with minimal sample preparation.^70^ Additionally, the researchers conducted an *in vitro* cellular uptake study to determine the *K*_d_ of the tracer using both LA-ICP-MS and fluorescent cytometry. The measured *K*_d_’s were 352 ± 141 nM by LA-ICP-MS and 245 ± 65 nM by fluorescent cytometry.

The results obtained by LA-ICP-MS and ICP-MS in the Vanhaecke’s work were consistent with each other and also in good agreement with those obtained by fluorescent and radioactive assays.^12^ For example, the evaluation of Ho content in tissue samples using ICP-MS revealed a distribution trend similar to that obtained using radioactive ^111^In. Additionally, the tumor-to-muscle ratio for Ho measured by ICP-MS was 5.85 ± 1.40, close to the value of 4.38 ± 1.51 measured using radioactive ^111^In.

**Figure 8:**
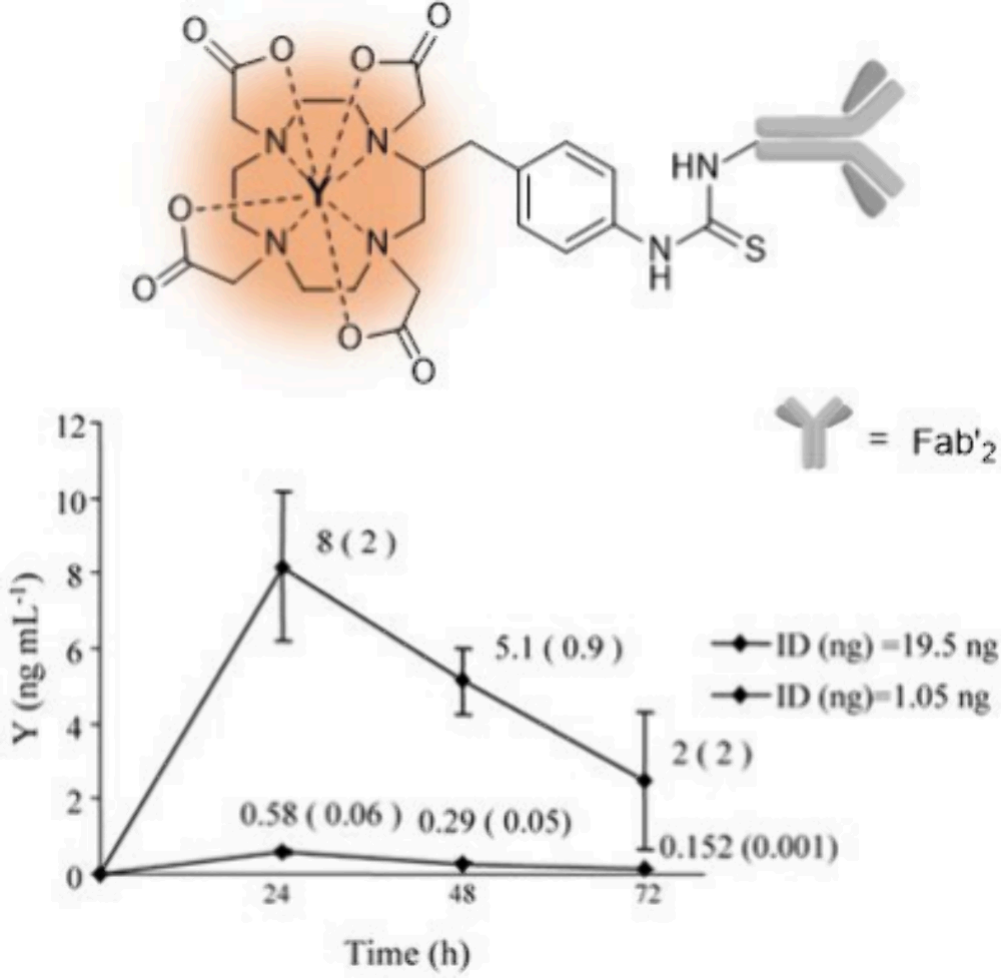
Renal clearance in normal mice treated with different dosages of ^nat^Y-Bz-DOTA-Fab’_2_ measured by ICP-MS as reported by Ciavardelli et al.^32^ Adapted with permission from Ciavardelli et al., An Inductively Coupled Plasma Mass Spectrometry Method for the Quantification of Yttrium-antibody Based Drugs Using Stable Isotope Tracing. *Rapid Commun. Mass Spectrom.***2007**, *21* (14), 2343–2350; copyright 2007 John Wiley & Sons.

As already mentioned, ten years prior to the work of Vanhaecke, Ciavardelli’s group investigated the renal clearance of a nonradioactive yttrium-labeled DOTA–mAb conjugate (^89^Y-Bz-DOTA-Fab’_2_) using ICP-MS (Figure 8).^32^ It is worth noting that radioisotopes of yttrium are well-known theranostic elements for use in radiopharmaceuticals and nuclear medicine (Table 1). Although the research from Ciavardeli et al. was the first example of using ICP-MS for pharmacokinetic studies of metal-conjugates and radiopharmaceutical models, it was limited only to urinary uptake and focused more on the analytical aspects of the method. Therein, the researchers optimized various operational parameters, including plasma power, gas flow rates, and sample introduction settings to enhance detection and minimize interference from the sample matrix. They analyzed urine samples from mice that were administered the ^nat^Y-labeled DOTA-conjugate with urine collection performed at time intervals of 0–24, 24–48, and 48–72 h postinjection. The injected dosages of 19.5 and 1.05 ng displayed similar patterns of urinary excretion in that both dosages exhibited similar proportional rates of yttrium clearance from the body (Figure 8), suggesting that the pharmacokinetic behavior of the antibody conjugate is consistent regardless of the administered dose. While this research describes a detailed ICP-MS-based bioassay, it does not provide any comparisons with results obtained via radioactive methods or any additional information regarding the pharmaceutical profile of their mAb conjugate.

The most significant advantage of ICP-MS over traditional radioanalytical methods in fundamental radiopharmaceutical research is, of course, the capability to conduct nonradioactive studies. Nevertheless, with abidance to mandatory regulations and safety considerations, ICP-MS-based assays can still be utilized to analyze radioactive probes where radioactivity cannot be avoided, e.g. in the case of technetium whose isotopes are all radioactive, and thus benefit from the other advantages of ICP-MS. An example is the 2024 report by Horstmann et al. where ^99^TcO_4_^–^ was quantified in patient urine samples using anion-exchange chromatography coupled to ICP-MS.^71^ The study, however, also mainly focused on instrumental settings and analytical methodology. While some clinically routine ^99m^Tc-based radiotracers, such as ^99m^Tc-MDP for bone scans, do not specifically target tumors like radiolabeled peptides or antibodies that bind to specific receptors, they do exhibit affinities for particular organs such as bones and the thyroid gland.^4,71^ In their work, Horstmann and colleagues measured the concentration of ^99^TcO_4_^–^ in untreated urine collected from a patient who had previously undergone scintigraphy with a ^99m^Tc-MDP radiotracer and validated the result against other methods including total reflection X-ray fluorescence and isobaric dilution analysis.

## Other Complementary Studies

**Figure 9:**
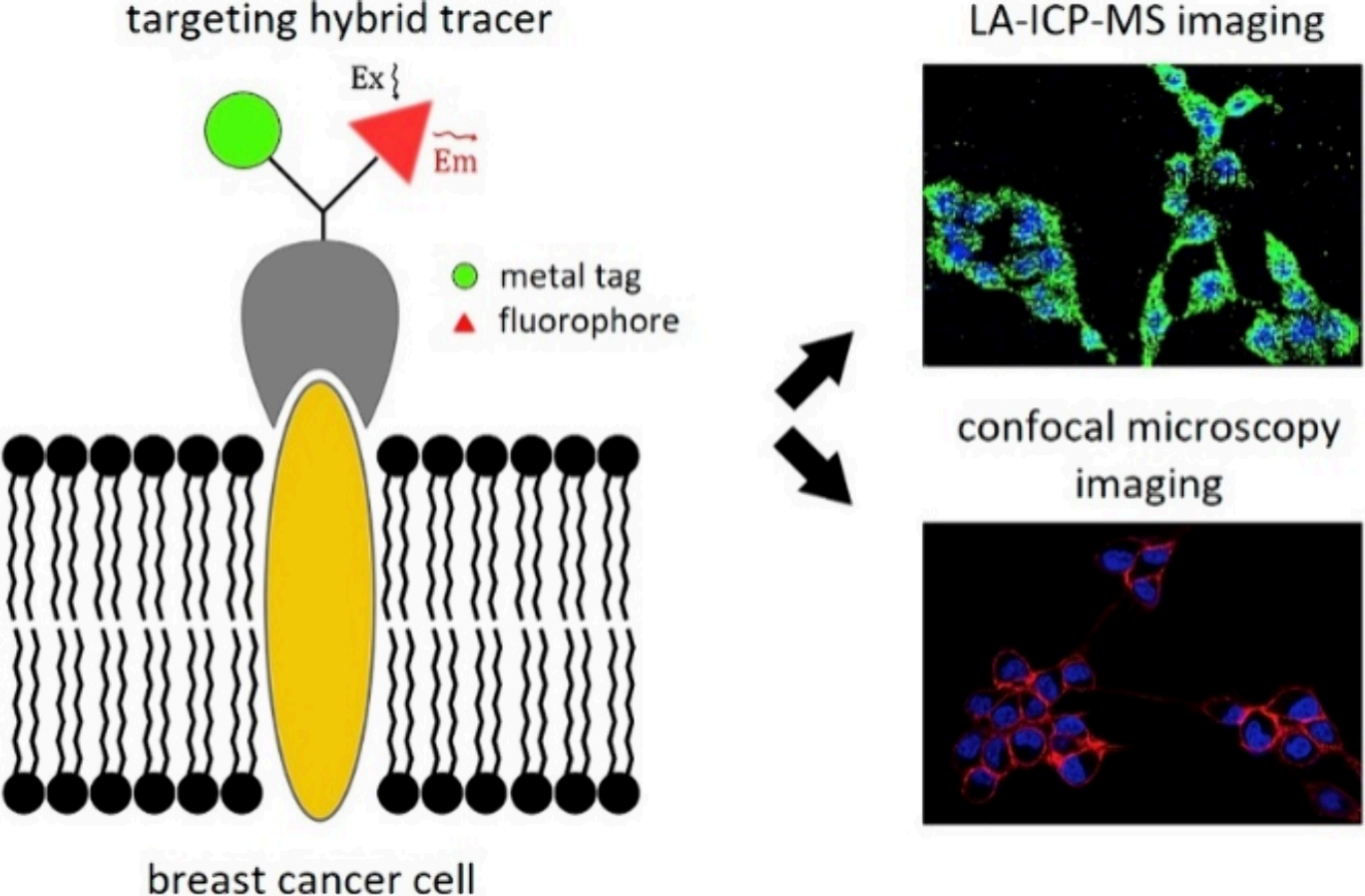
ICP-MS-based assay for single-cell analysis using targeting hybrid tracers to determine expression levels in breast cancer cells reported by Vanhaecke et al.^33^ Reproduced with permission from Vanhaecke et al., High-Resolution Imaging and Single-Cell Analysis via Laser Ablation-Inductively Coupled Plasma-Mass Spectrometry for the Determination of Membranous Receptor Expression Levels in Breast Cancer Cell Lines Using Receptor-Specific Hybrid Tracers. *Anal. Chim. Acta***2019**, *1074*, 43–53; copyright 2019 Elsevier Ltd.

There are various reports on the use of ICP-MS in single-cell analysis wherein researchers primarily describe the behavior of individual cells toward metal-based targeting conjugates, but obviously not the pharmaceutical profiles of these conjugates.^27,29,33,34^ Although the cellular assays established in such reports have potential applications in radiopharmaceutical and theranostic cancer research, the reports themselves do not discuss these applications.^13^ As an example, Vanhaecke et al. employed LA-ICP-MS to quantitatively determine receptor expression levels and reveal the 2D distribution of the corresponding proteins in breast cancer cell lines–specifically epidermal growth factor receptor (EGFR) and CXCR4–using hybrid tracers (Figure 9).^33^ Their tracers contained both a fluorophore and a metallic element (holmium, yttrium, or thulium), thereby enabling direct comparison between confocal fluorescence microscopy and ICP-MS-based assays for the quantification of receptor expression at the single-cell level. The results showed that LA-ICP-MS imaging could differentiate between cells based on their receptor expression levels. In addition, the study also demonstrated the complementary capabilities of confocal fluorescence microscopy and LA-ICP-MS. While confocal fluorescence microscopy provided high-resolution visualization of receptor locations, LA-ICP-MS offered precise, quantitative mapping of the tracers within the cells.

**Figure 10:**
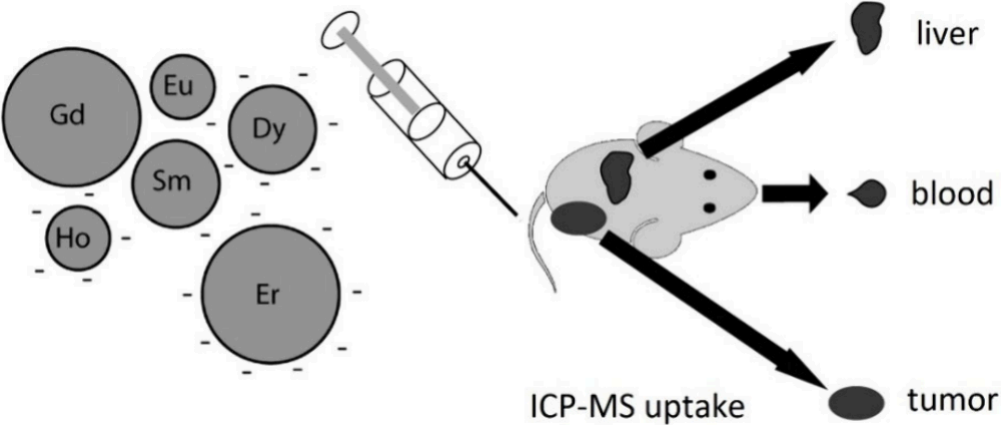
*In vivo* uptake reported by Crayton et al. of nanoparticles with the multiple elements present analyzed simultaneously by ICP-MS.^28^ Adapted with permission from Crayton et al., ICP-MS Analysis of Lanthanide-Doped Nanoparticles as a Non-Radiative, Multiplex Approach to Quantify Biodistribution and Blood Clearance. *Biomaterials***2012**, *33* (5), 1509–1519; copyright 2011 Elsevier Ltd.

There are also multiple studies that have explored the potential applications of metal-based nanoparticles in diagnostics and medical imaging utilizing ICP-MS as a key analytical technique.^28,72−74^ In some cases, the nanoparticles were conjugated to a bioactive vector to enhance their targeting properties.^75^ However, since the metal payload in such particles–whether conjugated to a vector or not–is located within a nanomaterial framework, they are not of particular interest in radiopharmacy and nuclear medicine; such constructs can complicate the radiolabeling and purification process of the final radiopharmaceutical. In typical radiopharmaceuticals, the metal payloads are held by a chelator within a conventional molecular system.^17^ Nonetheless, it is worth providing an example of using nanoparticles in ICP-MS-based tissue uptake to demonstrate the potential of this technique in diagnostics. For example, Crayton et al. prepared nanoparticles containing various lanthanides and conducted *in vivo* and *in vitro* investigations using ICP-MS (Figure 10).^28^ In their work, the authors discussed the potential applications of their nanoparticles for MRI imaging. Since their compound does not contain a targeting pharmacophore, it is classified as a nonspecific targeting probe. Their *in vitro* tests included stability tests in serum and cytotoxicity assays. For the *in vivo* investigation, they injected multielement formulations of the nanoparticles into subcutaneous tumor-bearing mice to evaluate their biodistribution, blood clearance, and tumor localization. Tissue samples were subsequently analyzed by ICP-MS to quantify the nanoparticles.

An interesting point raised by a reviewer in this context was the application of liposomes in the construction of theranostic metal-containing nanomaterials.^76,77^ Incorporating liposomes into their structures adds beneficial features to these theranostic agents, such as enhanced biostability and improved targeting efficiency. As an example, Jeon and co-workers introduced a theranostic bimetal-labeled nanomaterial comprising a ^64^Cu-NOTA-lipid radioconjugate and a dual-layered gold-liposome core for use in PET imaging.^76^ While such nanomaterials are ostensibly classified as nonspecific targeting agents, it is believed that they operate through a passive targeting mechanism whereby they accumulate to a greater extent in tumor tissues due to their leaky vascular nature. Hence, liposomes are basically more widely applicable in drug delivery than metallic nanoparticles. Although Jeon et al. did not employ ICP-MS to assess the bioactivity of their newly presented metal-liposome nanomaterial, such compounds can also be biologically investigated using ICP-MS-based assays.^77^

## Conclusion

A crucial question is whether it is always necessary, efficient, or meaningful to use significant resources to conduct radioactive experiments for research when nonradioactive techniques can yield comparable results. As many radiopharmaceutical research results may not be translated into clinical and real-world applications, it makes sense to avoid unnecessary use of radiochemistry in the early stages of research projects. Using nonradioactive techniques such as ICP-MS is a practical means for the pre-evaluation of metal-based conjugates and fundamental research on radiopharmaceuticals and imaging probes. In due course, radioactive approaches can subsequently be conducted to develop promising results obtained from ICP-MS studies. Due to the many issues with using radioactive compounds, we strongly urge researchers in the fields of radiopharmaceutical drug discovery and cancer theranostics to consider minimizing their use of radioactive compounds and explore alternative nonradioactive techniques, such as ICP-MS-based assays, in the early stages of their research projects. However, it is worth noting that nonradioactive techniques come with their own set of advantages and limitations and thus may not be applicable in all cases.

While ICP-MS techniques can be used in the clinical phase to assess theranostic agents—such as renal clearance and blood stability—current technologies do not allow it to serve as an alternative to nuclear imaging (e.g., PET scan). Nonradioactive clinical research on radiopharmaceutical models can be particularly useful for studying drug safety, dosimetry, and formulation. In preclinical and *ex vivo* research, ICP-MS techniques are especially beneficial for investigating organ distribution patterns and tumor targeting in animal models. As described in this Perspective, an increasing number of radiopharmaceutical researchers are utilizing ICP-MS-based assays in their work and we hope this trend will generate even greater interest in the field in the future.
